# Impact of hopelessness on migration intentions of nursing students: a path analysis

**DOI:** 10.1186/s12912-024-02667-5

**Published:** 2025-01-09

**Authors:** Soner Berşe, Emine Karacan, Pelin Zivdir Yeşılyurt, Zeynep Güngörmüş

**Affiliations:** 1 Department of Nursing, Faculty of Health Sciences, Gaziantep University, Gaziantep, Turkey; 2 Health Services Vocational School, Gaziantep Islam Science and Technology University, Gaziantep, Turkey; 3 Health Services Vocational School, Tarsus University, Mersin, Turkey; 4 Department of Nursing, Faculty of Health Sciences, Gaziantep Islam Science and Technology University, Gaziantep, Turkey

**Keywords:** Nursing, Student, Hopelessness, Brain drain

## Abstract

**Background:**

Brain drain refers to the migration of qualified professionals to developed countries in search of better living and working conditions, and has become a global concern, particularly in the healthcare sector. Migration of highly skilled nurses results in increased workload for the remaining nursing staff, limited access to quality healthcare services, and contributes to disparities in healthcare. Therefore, nursing students represent a critical demographic group for understanding the drivers of brain drain in the healthcare sector.

**Purpose:**

This study aimed to assess the levels of hopelessness experienced by nursing students in Turkey throughout their education and to examine the impact of hopelessness on their decisions to move abroad.

**Materials and methods:**

The study was conducted on 795 students enrolled in the nursing departments of two public universities in Gaziantep. Data were collected using a Demographic Data Form, the Beck Hopelessness Scale, and the Attitudes Towards Brain Drain Scale. The data were analyzed using the SPSS 24.0 statistical software.

**Results:**

57% of the nursing students considered moving abroad, with 20.2% preferring Germany. The primary reasons for their desire to migrate included poor living conditions (11.1%), economic problems (12.9%), unfavorable working conditions (8.3%), and educational challenges (5.4%). The findings highlight the significant role of future hopelessness in influencing the migration intentions of nursing students.

**Conclusion:**

More than half of Turkish nursing students consider moving abroad to seek better working conditions, higher living standards, financial gains and educational opportunities, with Germany being their top destination country. Despite low levels of hopelessness observed among the students, the tendency for migration persisted. A retention policy needs to be developed with strategic actions to prevent nurses from migrating to other countries.

**Clinical trial number:**

not applicable.

## Contributions to the literature

- This study explores the association between hopelessness and migration intentions among nursing students in Turkey.
- The findings highlight the significant role of future hopelessness in influencing the migration intentions of nursing students.
- The study provides essential insights for policymakers and educational institutions to develop strategies aimed at reducing brain drain by improving living and working conditions for nurses in Turkey.

## Introduction

The term “brain drain” refers to the migration of highly skilled individuals from developing countries to developed countries in search of better pay and working conditions, professional development and higher living standards, thereby contributing socially, culturally, and economically in the destination country [1, 2]. Currently, brain drain is rapidly increasing, and the nursing profession is significantly affected by this trend [3, 4].

The phenomenon of nurse migration actually emerged in the 1940s and showed a steady increase over time. Globally, the percentage of nurses working away from their country of birth increased from 5% in the 1970s to 13–37% in recent years [5, 6]. This means that approximately one in every eight qualified nurses practices in a country other than the one they were trained [7]. Factors pushing nurses towards moving abroad include increasing incidents of violence in healthcare settings, stressful working conditions, poor healthcare management, low wages, long working hours, and inadequate staffing levels. Additionally, poor living conditions, political instability and oppression, dangerous working conditions, economic hardships, war, lack of freedom of expression, feelings of insecurity and uncertainty about the future, and hopelessness related to not seeing any improvement in current conditions also accelerate nurse migration. Conversely, improved working conditions, access to advanced technology and education, career opportunities, new experiences, and the desire to be part of a medical team that offers greater autonomy and is highly respected are the pull factors that motivate nurse migration [6, 8].

Nurse migration can be associated with a number of benefits, such as aiding in the development of transnational connections and partnerships, gaining experience, knowledge and new skills, and sending remittances to their families, thereby driving economic growth in their home countries [9].

Brain drain, which implies a loss to the source country of vital skills and professional knowledge, is only relevant as a concept if linked with permanent migration. If the migrant nurses return to their home country, they will once again be a national resource [10]. However, when nurses settle in developed countries, it results in a free export of skilled human resources, representing the most significant cost of international migration [11]. Furthermore, the migration of qualified workforce increases the burden on remaining healthcare workers. This negatively affects the quality of patient care, limiting access to quality healthcare services for the community, and results in unequal distribution of the global health workforce. The WHO reported a global shortage of 9 million nurses and midwives, and projections for 2030 suggest that the situation will become even more concerning [11]. In OECD countries, there are 3.6 physicians, 8.8 nurses, and 4.4 beds per 1000 people. In Turkey, however, there are 2 physicians, 2.4 nurses, and 2.9 beds per 1000 people, placing Turkey just above Colombia and second to last among OECD countries [12]. The economic crisis ongoing since 2008, political instability following the 2016 coup attempt, and particularly the COVID-19 pandemic have influenced young nurses’ decisions to leave Turkey, raising concerns about potential future nurse shortages in the country. Accurate planning of healthcare services and a thorough understanding of the factors influencing the future size of the healthcare workforce are essential to mitigate nurse shortages in the future [6].

Previous studies involving nursing students in Turkey have mainly focused on the development of a scale to determine the viewpoints of nursing students about brain drain [13] and assessment of their attitudes towards migration [2, 6, 14, 15]. However, to the best of our knowledge, there are no studies examining the drivers of migration, with a specific focus on the association between feelings of hopelessness about the future and the tendency to migrate among nursing students in Turkey.

This study aimed to investigate the levels of hopelessness about the future among nursing students in relation to their intentions to migrate in a province of Turkey. By including students from two public universities, representing an integral part of the future healthcare workforce, this study sought to provide valuable insights for policymakers and nursing educators. The anticipated outcomes of this study are intended to extend beyond academic contributions. They are expected to inform strategic interventions aimed at reducing brain drain among nursing students. By exploring the association between hopelessness and the tendency to migrate, this research aimed to catalyze the development of targeted initiatives to retain talent within the local healthcare sector. This effort was undertaken not only to enhance nursing education but also to improve healthcare service planning, with the aim to contribute to the existing body of knowledge on this alarming issue.

## Materials and methods

### Study design

This study used a path analysis design to examine the association between hopelessness among nursing students and their intention to migrate.

### Study setting and timeline

The study was conducted between July 2022 and August 2023 at two public universities in Gaziantep.

### Study population and sample

The study population consisted of 1,219 students enrolled in the nursing departments of two public universities in Gaziantep. From this population, a sample of 795 nursing students, who were proficient in Turkish and agreed to participate voluntarily, was selected through random sampling. This approach was used to ensure that the findings would be generalizable and representative of the entire population.

### Data collection tools

#### Demographic data form

The demographic data form developed by the authors included questions related to key sociodemographic characteristics and migration intentions of the students. The variables covered in the form were gender, place of birth, nationality, grade level, and income level. Additionally, the form asked whether the students were considering moving abroad, their preferred country for migration, and reasons for their desire to migrate, such as poor living conditions, economic problems, unfavorable working conditions, and educational challenges. It also inquired whether students would want to migrate even if conditions improved in Turkey, as well as their disbelief in future improvements and their desire to explore new places and cultures.

#### Beck hopelessness scale (BHS)

The BHS developed by Beck et al. (1974) was designed to assess individuals’ level of hopelessness about the future [16]. The validity and reliability of the Turkish version of the BHS were demonstrated by Durak (1994) [17]. The scale consists of 20 items, with an answer key of 11 “yes” and 9 “no” responses. A score of 1 point is assigned if the response is “yes” for questions 2, 4, 7, 9, 11, 12, 14, 16, 17, 18, and 20; or if the response is “no” for questions 1, 3, 5, 6, 8, 10, 13, 15, and 19. Otherwise, the response is assigned a score of 0 points. The scale comprises three subscales: feelings about the future (items 1, 6, 13, 15, 19), loss of motivation (items 2, 3, 9, 11, 12, 16, 17, 20), and future expectations (items 4, 7, 8, 14, 18). Total possible scores range from 0 to 20. Higher scores indicate greater levels of hopelessness. Durak (1994) reported a Cronbach’s alpha value of 0.85 for the scale [17]. In our study, the Cronbach’s alpha values were 0.81, 0.75, 0.80 for the subscales of feelings about the future, loss of motivation, future expectations respectively, and 0.86 for the entire scale.

### Attitudes towards brain drain scale (ABDS)

The 16-item Likert-type scale, developed by Öncü et al. (2018) was designed to assess nursing students’ attitudes towards brain drain. The ABDS is a one-dimensional scale with two components: Pull Factors (1, 2, 3, 4, 5, 6, 8, 10, 12, 14, 15, 16) and Push Factors (7, 9, 11, 13). Items 3 and 15 are reverse-coded. Total possible scores range from 16 to 80, with higher scores indicating greater inclination towards migration. The Cronbach’s alpha values for the entire scale and for the Pull and Push Factors subscales were 0.91, 0.88 and 0.86, respectively [13]. In the current study, the Cronbach’s alpha values for the Pull Factors, Push Factors, and total scale were 0.90, 0.92, and 0.93, respectively.

### Data analysis

Data analysis was performed using IBM^®^ SPSS 24.0 software. Descriptive statistics were reported as number and percentage for the students’ demographic data and mean and standard deviation for the total scale scores. Independent samples t-test and ANOVA were used for comparisons between the total scale scores and the demographic characteristics. The significance level was set at *p* < 0.05. Additionally, path analysis was conducted to examine the impact of hopelessness on brain drain.

### Ethical considerations

Ethical approval was obtained from the Institutional Review Board of Gaziantep Islam Science and Technology University (2022/121- 121.17.10). Permission to conduct the study was obtained from both universities. The students signed informed consent before initiation of the study. The study was conducted in accordance with the ethical principles laid out in the Declaration of Helsinki.

## Results

The sociodemographic characteristics of the nursing students and their perspectives on migration are presented in Table 1. 57% of the students indicated that they are considering moving abroad. Among the reasons cited for this decision, the most common were economic problems (12.9%) and poor living conditions (11.1%), while educational challenges (5.3%) and disbelief in any improvement in the future (5.5%) were less frequently mentioned.

**Table 1:** Demographic characteristics of nursing students

Demographic Characteristics (*n* = 796)		*n*	%
**Age**,** years (mean ± SD)**: 20.55 ± 1.68			
Gender	Male	163	20.5
	Female	633	79.5
Place of birth	South of Turkey	509	63.9
	East of Turkey	56	7
	North of Turkey	22	2.8
	West of Turkey	170	21.4
	Abroad	39	4.9
Nationality	Turkish	761	95.6
	Foreign (Syria, Iran, Iraq, Norway, Turkmenistan, Saudi Arabia, Romania)	35	4.4
Grade	1st year	338	42.5
	2nd year	275	34.5
	3rd year	127	16
	4th year	56	7
Income level	Income lower than expenses	202	25.4
	Income equal to expenses	493	61.9
	Income higher than expenses	101	12.7
**Considering moving abroad**	Yes	454	57
	No	342	43
Preferred country for migration*	Germany	161	20.2
	USA	62	7.8
	United Kingdom	70	8.8
	Canada	52	6.5
	Other (Italy, Sweden, France)	99	12.4
	Does not wish to migrate/Undecided	353	44.3
**Reasons for the desire to move abroad**			
**Poor living conditions**	Yes	88	11.1
	No	708	88.9
**Economic problems**	Yes	103	12.9
	No	693	87.1
**Unfavorable working conditions**	Yes	66	8.3
	No	730	91.7
**Educational challenges**	Yes	42	5.4
	No	754	94.6
**Desire to move to abroad even if conditions improve in Turkey**	Yes	156	19.6
	No	640	80.4
**Disbelief in any improvement in the future**	Yes	44	5.5
	No	752	94.5
**To explore new places and meet people from different cultures**	Yes	54	6.8
	No	742	93.2

*Participants may have selected multiple options

The mean score for the subscale of feelings about the future was significantly higher among the students of Turkish nationality, those with lower income, those considering moving abroad, and citing reasons such as poor living conditions, unfavorable work environment, and economic factors for their migration intentions (*p* < 0.05) (Table 2).

**Table 2:** Comparison of demographic characteristics with total and mean subscale scores of the beck hopelessness scale (BHS) and the attitudes towards brain drain scale (ABDS)

Demographic Characteristics (*n* = 795)	BHS and its subscales				ABDS and its subscales		
	Feelings about the future	Loss of motivation	Future expectations	Total Score	Pull Factors	Push Factors	Total Score
**Gender**							
Male	1.85 ± 1.74	2.78 ± 2.25	2.45 ± 1.58	7.94 ± 5.03	42.93 ± 9.68	16.05 ± 3.84	59.07 ± 12.96
Female	2.05 ± 1.84	3.53 ± 2.26	2.84 ± 1.62	9.23 ± 5.16	46.28 ± 8.94	16.92 ± 3.51	63.38 ± 11.64
**t/p**	1.221/0.223	3.80/0.00*	2.76/0.00*	2.90/0.00*	3.99/0.00*	2.62/0.00*	3.86/0.00*
**Place of birth**							
South of Turkey	1.91 ± 1.77	2.93 ± 2.25	2.58 ± 1.58	8.26 ± 5.06	43.22 ± 9.80	16.13 ± 3.90	59.41 ± 13.16
East of Turkey	1.89 ± 1.69	2.69 ± 2.10	2.21 ± 1.51	7.42 ± 4.37	43.69 ± 8.97	16.39 ± 3.82	60.08 ± 12.37
North of Turkey	2.27 ± 1.95	4.36 ± 2.71	3.00 ± 1.57	10.63 ± 6.08	48.50 ± 8.04	18.13 ± 2.98	66.63 ± 10.78
West of Turkey	2.01 ± 1.80	2.83 ± 2.39	2.57 ± 1.71	8.30 ± 5.44	44.35 ± 9.65	16.38 ± 3.69	60.91 ± 12.68
Abroad	1.12 ± 1.36	3.05 ± 1.77	1.92 ± 1.22	6.76 ± 3.41	42.61 ± 8.34	15.58 ± 2.93	58.94 ± 9.23
**F/p**	2.31/0.05	2.44/0.04*	2.64/0.03*	2.41/0.04*	1.98/0.09	1.85/0.11	2.03/0.08
**Nationality**							
Turkish	1.93 ± 1.77	2.91 ± 2.29	2.56 ± 1.61	8.24 ± 5.14	43.61 ± 9.71	16.25 ± 3.81	59.98 ± 12.91
Foreign	1.28 ± 1.50	3.48 ± 1.88	2.02 ± 1.17	7.45 ± 3.57	43.68 ± 7.62	15.71 ± 3.52	59.40 ± 10.65
**t/p**	2.46/0.01*	1.73/0.09	2.57/0.01*	1.24/0.22	0.04/0.96	0.82/0.40	0.26/0.79
Grade							
1st year	2.01 ± 1.76	3.29 ± 2.26	2.65 ± 1.56	8.81 ± 4.87	44.04 ± 8.80	16.30 ± 3.65	60.43 ± 11.79
2nd year	1.79 ± 1.76	2.54 ± 2.22	2.31 ± 1.57	7.44 ± 5.08	43.48 ± 10.68	16.24 ± 3.94	59.95 ± 13.97
3rd year	2.03 ± 1.76	3.11 ± 2.31	2.88 ± 1.63	8.92 ± 5.24	43.21 ± 9.89	16.01 ± 3.98	59.22 ± 13.52
4th year	1.46 ± 1.77	2.32 ± 2.18	2.12 ± 1.66	6.64 ± 5.28	42.57 ± 8.36	16.23 ± 3.58	58.80 ± 13.30
**F/p**	2.19/0.08	7.22/0.00*	5.76/0.00*	6.42/0.00*	0.53/0.65	0.17/0.91	0.44/0.72
**Income level**							
Income lower than expenses	2.60 ± 1.82	3.75 ± 2.36	3.09 ± 1.55	10.48 ± 5.01	45.32 ± 9.20	16.83 ± 3.64	62.45 ± 11.83
Income equal to expenses	1.67 ± 1.69	2.68 ± 2.18	2.36 ± 1.57	7.48 ± 4.93	43.08 ± 9.24	16.06 ± 3.74	59.21 ± 12.42
Income higher than expenses	1.61 ± 1.63	2.57 ± 2.19	2.27 ± 1.56	7.17 ± 4.61	42.78 ± 11.78	15.83 ± 4.24	58.61 ± 15.75
**F/p**	22.71/0.00*	18.05/0.00*	17.06/0.00*	29.22/0.00*	4.31/0.01*	3.55/0.02*	5.26/0.00*
**Considering moving abroad**							
Yes	2.12 ± 1.77	3.11 ± 2.32	2.74 ± 1.63	8.86 ± 4.09	47.10 ± 7.94	17.39 ± 3.06	64.69 ± 10.08
No	1.61 ± 1.71	2.70 ± 2.19	2.26 ± 1.50	7.33 ± 4.79	38.98 ± 9.73	53.67 ± 13.36	53.67 ± 13.36
**t/p**	4.02/0.00*	2.50/0.01*	4.21/0.00*	4.22/0.00*	12.59/0.00*	10.22/0.00*	12.75/0.00*
**Desire to move abroad due to poor living conditions**							
Yes	2.51 ± 1.85	3.38 ± 2.51	2.97 ± 1.63	9.93 ± 5.32	48.90 ± 6.94	18.04 ± 2.61	66.95 ± 9.18
No	1.82 ± 1.74	2.88 ± 2.24	2.48 ± 1.58	7.99 ± 5.01	42.95 ± 9.71	16.00 ± 3.86	59.08 ± 12.94
**t/p**	3.43/0.00*	1.95/0.05	2.73/0.00*	3.39/0.00*	7.20/0.00*	6.48/0.00*	7.19/0.00*
**Desire to move abroad due to economic problems**							
Yes	2.41 ± 1.80	3.30 ± 2.23	3.06 ± 1.67	9.74 ± 5.10	47.99 ± 7.77	17.71 ± 2.82	65.67 ± 10.17
No	1.82 ± 1.75	2.88 ± 2.28	2.46 ± 1.57	17.97 ± 5.04	42.97 ± 9.71	16.01 ± 3.87	59.10 ± 12.96
**t/p**	3.17/0.00*	1.72/0.08	3.62/0.00*	3.31/0.00*	5.86/0.00*	5.41/0.00*	5.88/0.00*
**Desire to move abroad due to unfavorable working conditions**							
Yes	2.34 ± 1.72	2.96 ± 2.14	2.60 ± 1.69	8.89 ± 5.30	47.83 ± 8.83	17.60 ± 3.40	65.90 ± 10.49
No	1.86 ± 1.76	2.93 ± 2.29	2.53 ± 1.59	8.14 ± 5.06	43.23 ± 9.61	16.10 ± 3.80	59.42 ± 12.99
**t/p**	2.13/0.03*	0.11/0.91	0.35/0.72	1.14/0.25	3.74/0.00*	3.08/0.00*	3.92/0.00*
**Desire to move abroad due to educational challenges**							
Yes	1.83 ± 1.73	3.02 ± 2.27	2.45 ± 1.65	7.90 ± 5.35	48.02 ± 7.23	17.16 ± 3.26	65.19 ± 10.05
No	1.90 ± 1.77	2.93 ± 2.27	2.54 ± 1.59	8.22 ± 5.07	43.37 ± 9.69	16.18 ± 3.82	59.66 ± 12.90
**t/p**	0.26/0.78	0.24/0.80	0.36/0.71	0.39/0.69	3.06/0.00*	1.64/0.10	2.72/0.00*
**Desire to move abroad even if conditions improve in Turkey**							
Yes	1.80 ± 1.77	2.96 ± 2.36	2.49 ± 1.54	8.03 ± 5.16	46.46 ± 9.47	17.19 ± 3.29	63.84 ± 12.05
No	1.92 ± 1.76	2.93 ± 2.25	2.55 ± 1.61	8.24 ± 5.07	42.92 ± 9.54	15.99 ± 3.87	59.01 ± 12.83
**t/p**	0.81/0.41	0.13/0.89	0.39/0.69	0.46/0.64	4.15/0.00*	3.57/0.00*	4.26/0.00*
**Desire to move abroad because of disbelief in any improvement**							
Yes	2.20 ± 1.78	3.22 ± 2.41	2.86 ± 1.66	9.25 ± 5.36	51.90 ± 5.27	18.63 ± 2.20	70.54 ± 8.22
No	1.88 ± 1.76	2.92 ± 2.27	2.51 ± 1.59	8.14 ± 5.06	43.13 ± 9.57	16.09 ± 3.82	59.33 ± 12.77
**t/p**	1.15/0.24	0.86/0.38	1.38/0.16	1.40/0.16	8.70/0.00*	7.07/0.00*	8.46/0.00*
**Desire to move abroad to explore new places and meet different people**							
Yes	1.46 ± 1.70	2.24 ± 2.29	2.22 ± 1.57	6.48 ± 5.20	47.29 ± 7.98	17.38 ± 2.68	64.68 ± 10.45
No	1.93 ± 1.76	2.99 ± 2.72	2.56 ± 1.60	8.33 ± 5.05	43.34 ± 9.69	16.14 ± 3.85	59.61 ± 12.91
**t/p**	1.90/0.05	2.34/0.01*	1.50/0.13	2.59/0.01*	2.92/0.00*	3.16/0.00*	2.81/0.00*

* *p* < 0.05

The mean score for the subscale of loss of motivation was significantly higher among female participants, students from the northern parts of Turkey, the first-year students, those with lower income, those considering moving abroad, and those who expressed disinterest in exploring new places and meeting individuals from different cultures (*p* < 0.05) (Table 2).

The mean score for the subscale of future expectations was significantly higher among female participants, students from the northern parts of Turkey, Turkish citizens, the third-year students, those with lower income, those considering moving abroad, and those who were motivated to move abroad due to poor living conditions and economic factors (*p* < 0.05) (Table 2).

The total BHS score was significantly higher among female participants, students from the northern parts of Turkey, the third-year students, those with lower income, those considering moving abroad, those motivated to move abroad due to poor living conditions, those not citing economic factors as a reason to move abroad, and those who were disinterested in exploring new places and meeting people from different cultures (*p* < 0.05) (Table 2).

The total score and the score for Pull Factors subscale of the ABDS were significantly higher among female participants, the students with lower income, those considering moving abroad, those with a desire to move abroad due to poor living and working conditions, educational and economic issues, those who believed that nothing will get any better even if conditions improve in Turkey, and those interested in moving abroad to explore new places and meet people from different cultures (*p* < 0.05) (Table 2).

The Push Factors subscale score was significantly higher among female participants, those with lower income, the students who did not consider moving abroad permanently, those with a desire to move abroad due to poor living and working conditions, and economic issues, those who believed that nothing will get any better even if conditions improve in Turkey, and those interested in moving abroad to explore new places and meet people from different cultures (*p* < 0.05) (Table 2).

The mean total and subscale scores were as follows: Beck Hopelessness Scale (BHS) total score ranged from 0 to 20, with a mean of 8.2 ± 5.0. The subscales of BHS were as follows: Feelings about the future (range: 0–5) with a mean score of 1.90 ± 1.76; Loss of motivation (range: 0–7) with a mean score of 2.9 ± 2.2; and Future expectations (range: 0–5) with a mean score of 2.5 ± 1.6.

For the Attitudes Towards Brain Drain Scale (ABDS), the total score ranged from 16 to 80, with a mean of 59.9 ± 12.8. The subscale scores were as follows: Pull Factors (range: 12–60) with a mean of 43.6 ± 9.6; and Push Factors (range: 4–20) with a mean of 16.2 ± 3.7 (Table 2).

In this study, the relationship between hopelessness and migration intentions (brain drain) among nursing students was investigated using path analysis. The analysis revealed a significant direct association between hopelessness, measured by the Beck Hopelessness Scale (BHS), and migration intentions, measured by the Attitudes Towards Brain Drain Scale (ABDS) (*r* = 0.246, *p* < 0.05) (Table 3). The regression equation derived from the model is as follows:

**Table 3:** Path analysis to determine the effect of hopelessness on Brain Drain

Variables	Unstandardized Coefficients		Standardized Coefficients	t	*p*
	B	Std. Error	Beta		
Constant variable	54.863	0.837		65.559	0.001
Total BHS Score	0.621	0.087	0.246	7.163	0.001

Brain Drain = 54.86 + 0.621 * Hopelessness.

This indicates that for every one-unit increase in the BHS score, the ABDS score increases by 0.621 points, highlighting a substantial positive impact of hopelessness on the tendency to migrate. The standardized coefficient (β = 0.246, *p* < 0.05) further supports this significant association.

## Discussion

In this study investigating the level of hopelessness among nursing students from two public universities in Gaziantep, Turkey and its impact on their intentions to migrate, it was found that a significant portion of the students were considering moving abroad, with a notable preference for Germany. Economic factors, poor working conditions, and educational challenges were identified as the main reasons for the desire to move abroad. Furthermore, the students showed low levels of hopelessness and a high tendency for migration.

Healthcare professionals are among the occupational groups that are most affected by brain drain. The international migration of nurses from developing countries to developed countries has accelerated (International Organization for Migration, 2021). The increase in nurse migration can be attributed to various factors, including globalization, the removal of borders between countries, advancements in transportation and communication, and technological developments, as well as migration-promoting policies of developed countries driven by changing economic and political structures [13]. Although there is evidence indicating an increase in nurse migration in Turkey in recent years, few studies are available [2, 6, 13–15, 18]. Given the significant shortage of qualified healthcare workers in Turkey, it is essential to gather evidence-based data and develop future human resource strategies.

A noteworthy finding of the present study was that, although the female nursing students had higher expectations for the future compared to their male counterparts, they also exhibited higher levels of motivation loss and hopelessness. This suggests that female students are more likely to be affected by the push factors in Turkey and the pull factors in other countries which may be influential in the decision to migrate. This observation is in line with previous research indicating that female nursing students tend to experience higher levels of hopelessness [19] and a stronger inclination to move abroad [20]. In contrast, some studies have reported higher levels of hopelessness among male students [2, 21], while others found no gender-based difference in the decision to migrate [22, 23]. Although nursing has traditionally been perceived as a female-dominated profession in Turkey, it is increasingly being chosen by men, albeit in smaller numbers [24]. In Turkey, with its patriarchal social structures and male dominance, women often find themselves in secondary positions across various domains including education, the workplace, politics, household roles, interpersonal relationships, and public sphere [25]. Therefore, we think that female nursing students in Turkey may be negatively affected by gender inequality, leading to feelings of hopelessness about the future and a greater inclination to migrate due to not having equal opportunities as their male counterparts.

In the present study, the nursing students of Turkish nationality reported negative feelings and pessimism about the future. Interestingly, the students coming from the northern regions of Turkey had higher expectations for the future but showed higher levels of motivation loss and hopelessness compared to those from other regions of the country. Similarly, in a study, Turkish students were found to have higher levels of hopelessness compared to German students [26]. These results suggest that economic conditions, development levels, and cultural factors in different regions and countries may affect young people’s future prospects. We think that social, economic, educational challenges, as well as unemployment may diminish the hopes of young people and drive them toward migration in search of better opportunities.

In this study, while the first-year nursing students exhibited lower levels of motivation regarding their future, the third-year nursing students showed higher expectations but also higher levels of hopelessness about the future. However, in previous studies, it was found that second-year students [2] and fourth-year students [24] had higher levels of hopelessness. It may be assumed that first-year nursing students, being at the outset of their education with no experience, may not have fully developed prospects for the future compared to other students. The higher levels of expectation and hopelessness observed among the third-year students may reflect their greater awareness of current problems in the healthcare system, as well as their maturation and greater exposure to the challenges and realities of their educational and cultural environment.

In the current study, it was anticipated that the nursing students with lower income would exhibit higher levels of hopelessness and a stronger desire to move abroad. The rapid increase in the number of universities in Turkey, the growing number of graduates, the competitive job market with thousands of young people vying for limited positions, and prevailing unemployment issues suggest that those students facing economic hardships may feel hopeless. Consequently, they might seek opportunities in wealthy countries that offer better prospects and improved conditions.

In this study, 57% of the nursing students considered moving abroad permanently. These students displayed heightened concerns, expectations, loss of motivation, and hopelessness about the future, which triggered a stronger inclination towards migration. Similarly, studies on nursing students reported mild levels of hopelessness [2] and high scores on attitudes towards migration [6, 8, 14, 18]. Consistently, other studies from Turkey found that 66.3% [18] and 73.3% [8] of nursing students expressed a desire to work abroad after graduation. Studies on nursing students in other countries also indicate a similar trend, with 70% [27] and 91.3% [28] of the students intending to migrate. We think that nursing students in Turkey aspire to live in countries that offer better opportunities due to the poor working conditions, low wages, increased incidents of violence in healthcare settings, lack of policies to prevent workplace violence, labor rights violation, mobbing, feelings of devaluation, recurrent economic crises, lack of meritocracy, and political instability in the country. In line with our findings, in a study from Turkey examining the drivers of migration among nurses, the three most important reasons cited by the nurses were poor economic conditions, negative work environment, and low social value and prestige of the profession [29].

Migration results from a complex interplay of various push and pull factors. Push factors are those that influence individuals’ decisions to leave their own country, often related to unfavorable conditions in their current environment. On the other hand, pull factors are those that draw individuals to another country, such as job opportunities, better living and working conditions, opportunities for professional advancement, and a promise of freedom from religious or political persecution. Studies have reported that for nurses, the primary push factors include poor living conditions, economic hardships, security issues, political problems, oppression, wars, limited employment opportunities, insecurity, and weak healthcare management, and major pull factors include a stable life, family security, the need for skilled labor, and education, job, and career opportunities [13, 30, 31]. In this study, the students expressing a desire to move abroad due to concerns about living conditions, financial problems, working conditions, and educational issues showed high scores on the ABDS Pull and Push Factors subscales as well as a strong tendency to migrate. This finding aligns with the data from a previous study which reported that 59.5% of nurses considered migrating for education, 40.5% for better job opportunities, and 21.6% for a higher standard of living [32]. In addition, the desire to migrate even if conditions in Turkey improve, lack of belief that conditions will ever improve, and the desire to migrate to explore new places and meet people from different cultures were the most common reasons for considering migration among the nursing students. More recently, discouraging work conditions such as increased violence against healthcare workers and the excessive workload at the time of the pandemic seem to have reinforced the nursing students’ inclination towards migration.

Among the nursing students participating in this study, 20.2% expressed a preference to move to Germany. In contrast, a study by Nguyen et al. found that 59% of nursing students desired to work in the USA and 49% in the United Kingdom [27]. According to the UNESCO (United Nations Educational, Scientific and Cultural Organization), the top destination countries that receive the most immigrants include the USA, the UK, Germany, France, Australia, Japan, Russia, Spain, Belgium, Canada, and Italy [33]. The preference for Germany among the students may be attributed to several factors. Germany offers more favorable working conditions and wages for nurses compared to Turkey. Also, there is significant promotion of nursing job opportunities in Germany through internet and social media channels. Germany’s population is getting older, and consequently there is a high demand for nurses. Also, Germany is the top destination for migration due to its high-quality universities, advanced research centers, and extensive economic and cultural opportunities. As noted by Karaca and Yurttaş, the preference for Germany among healthcare workers may be influenced by the possibility that they would be subjected to less discrimination because of the large Turkish population living there [34].

### Limitations of the study

This study has several limitations that should be considered when interpreting the findings. The research was conducted with nursing students from two public universities in Gaziantep, limiting the generalizability of the results to students from other regions or institutions. The extended data collection period, spanning from July 2022 to August 2023, was necessary due to the challenges posed by a major earthquake in the study region. This timeline allowed the researchers to reach displaced participants and ensure a representative sample, but it may have introduced variability in external factors affecting the participants’ responses. Additionally, the researchers’ employment at the universities where data were collected could have influenced participants’ responses, despite measures such as anonymity and independent data collection being implemented to mitigate bias.

The cross-sectional design of the study provides a snapshot of the participants’ attitudes and experiences at a single point in time, limiting the ability to assess changes over time. Future research with a longitudinal approach could offer a deeper understanding of the dynamics between hopelessness and migration intentions. Furthermore, as the study relied on self-reported data, there is potential for social desirability bias or inaccuracies in responses, although efforts were made to ensure confidentiality and encourage honest reporting. Despite these limitations, the findings contribute valuable insights into the factors influencing nursing students’ migration intentions and provide a basis for future research and policy development.

## Conclusion and future directions

More than half of Turkish nursing students consider moving abroad to seek better working conditions, higher living standards, financial gains and educational opportunities, with Germany being their top destination country. In spite of low levels of hopelessness observed among the students, the tendency for migration persisted. These results highlight the need for policymakers and educational institutions to develop strategies and practices aimed at reducing migration tendencies of nursing students by focusing on improving nurses’ living and working conditions in Turkey.

In this context, the following actions are proposed:

### Financial incentives

- Adjusting nurse salaries to fair and competitive levels in line with international standards and the cost of living.
- Enhancing working conditions, reducing working hours to reasonable levels, and ensuring regular and equitable compensation for overtime.

### Enhancing education and career opportunities

- Improving the quality of nursing education and expanding opportunities for continuous education and professional development.
- Clearly defining career advancement steps and implementing supportive policies for career development.

### Improving the work environment

- Preventing violence in healthcare settings and ensuring a safe working environment for nurses.
- Enhancing the reputation of the nursing profession in the community and fostering a positive public perception of nursing.

The implementation of these recommendations could enhance the attractiveness of the nursing profession in Turkey, better address the demand for quality healthcare services, and mitigate the rates of brain drain.

## Data Availability

The datasets generated and/or analyzed during the current study are available from the corresponding author on reasonable request.
