# Correction: Impact of hopelessness on migration intentions of nursing students: a path analysis

**DOI:** 10.1186/s12912-025-02734-5

**Published:** 2025-01-23

**Authors:** Soner Berşe, Emine Karacan, Pelin Zivdir Yeşılyurt, Zeynep Güngörmüş

**Affiliations:** 1 Department of Nursing, Faculty of Health Sciences, Gaziantep University, Gaziantep, Turkey; 2 Health Services Vocational School, Gaziantep Islam Science and Technology University, Gaziantep, Turkey; 3 Health Services Vocational School, Tarsus University, Mersin, Turkey; 4 Department of Nursing, Faculty of Health Sciences, Gaziantep Islam Science and Technology University, Gaziantep, Turkey

**Correction to: BMC Nurs 24**,** 29 (2025)**

10.1186/s12912-024-02667-5

Following the publication of the original article [1], the author group identified errors in their affiliations.

The affiliation details for Soner Berşe were incorrectly given as “Health Services Vocational School, Gaziantep Islam Science and Technology University, Gaziantep, Turkey” but should have been “Department of Nursing, Faculty of Health Sciences, Gaziantep University, Gaziantep, Turkey”.

The affiliation details for Emine Karacan were incorrectly given as “Health Services Vocational School, Tarsus University, Mersin, Turkey” but should have been “Health Services Vocational School, Gaziantep Islam Science and Technology University, Gaziantep, Turkey”.

The affiliation details for Pelin Zivdir Yeşılyurt were incorrectly given as “Department of Nursing, Faculty of Health Sciences, Gaziantep University, Gaziantep, Turkey” but should have been “Health Services Vocational School, Tarsus University, Mersin, Turkey”. In addition, the author’s family name was corrected from “Yeşılyurt” to “Zivdir Yeşılyurt”.

The original article [1] has been updated.
